# Risk factors of pin tract infection during bone transport using unilateral external fixator in the treatment of bone defects

**DOI:** 10.1186/s12893-021-01384-z

**Published:** 2021-10-26

**Authors:** Kai Liu, Alimujiang Abulaiti, Yanshi Liu, Feiyu Cai, Peng Ren, Aihemaitijiang Yusufu

**Affiliations:** Department of Trauma and Microreconstructive Surgery, The First Affiliated Hospital of Xinjiang Medical University, Urumqi, 830054 Xinjiang China

**Keywords:** Bone transport, Ilizarov technique, External fixator, Infection, Pin tract

## Abstract

**Background:**

The bone transport using the unilateral external fixator, one of the Ilizarov techniques, is widely practiced in lower limb reconstructive surgery. Pin tract infection (PTI), one of most common complication, has become the important postoperative problems which plague clinicians gradually.

**Methods:**

A group of 130 patients who received bone transport surgery for tibia or femur defects using the unilateral external fixation (Orthofix limb reconstruction system, Verona, Italy) and met the inclusion criteria were selected for the study from 2015 to 2019. Regular pin tract care was performed twice a day, and the conditions of the pin tract were evaluated by the same observer using clinical appearance criteria. The Saw’s classification of PTI was used to assess the condition around screws. After the data were significant by the T-test or Pearson’s Chi-square test analyzed, odds ratios were calculated using logistic regression tests to describe factors associated with the diagnosis of PTI.

**Results:**

Ninety-one males and thirty-nine females with a mean age of 43 years (range 28–58 years) were included in this cohort. 7816 observations were documented from 12 to 36 months, and 58 cases (44.6%) of PTI (thirty-nine cases in grade 1, 17 cases in grade 2, and 2 cases in grade 3). The top five risk factors were agricultural work (OR 1.86, CI 0.94–2.39), non-urban living (OR 1.75, CI 1.24–3.26), male (OR 1.71, CI 1.02–2.31), smoking (OR 1.53, CI 0.76–1.89), and diabetes (OR 1.26, CI 1.12–2.64). No long-term sequelae were observed at the latest clinical visit.

**Conclusion:**

Occupation, gender, living environment (non-urban), smoking, and diabetes were the top five significant risk factors for PTI in the period of bone transport using unilateral external fixation. Awareness of predictable risk factors of PTI is beneficial to avoid or early detect the severe complications which can affect the effectiveness.

## Background

The bone transport using the unilateral external fixator, one of the Ilizarov techniques, has been a practical way to reconstruct lower limb bone defects caused by trauma, debridement of osteomyelitis, and tumor resection [1–9]. Its complications, however, have also attracted the attention of scholars gradually, especially pin tract infection (PTI). To our knowledge, the rate of PTI has been reported to vary from 6 to 96.6% [10]. A study of 88 patients about the incidence of PTI during limb lengthening using external fixation was published by Antoci et al. [11], concluded that the PTI rate was 96.6%. And a retrospective study of 163 patients who underwent retrograde percutaneous pinning of the distal femur for osteotomy or physeal fracture was conducted by Murgai e al. [12] and reported the incidence of PTI was 6.7%. The previous studies commonly focused on the treatments of PTI and percentages of patients with complications, concentrated on the risk factors less. Yet, a clear understanding of the risk factors causing PTI will reduce the waste of medical resources more effectively and the psychological burden of patients, in comparison to feasible remedies. It is also noteworthy that external fixation removal, recurrence of osteomyelitis, or even failure of the bone transport procedure may be caused by PTI if not treated in a proper and timely manner [13–15].

PTI may be caused by a combination of factors related to physical health and external fixator. The direct way between the bone and the outside environment was established by the natural characteristics of the external fixator, which created favorable conditions for microbial breeding around the pin tract. Factors associated with a higher risk of pin site infection were reported by a previous study [1, 10, 14, 16–18], including aging, chronic intrinsic comorbidities (diabetes, rheumatoid arthritis, and other collagen vascular diseases), and body immune status, unhealthy habits (obesity, smoking, and alcohol) and the usage of steroid hormonal agents. Besides, the different types of external fixators, locations of pin fixation, and frequent joint motion may increase the risk of PTI. Antoci et al. [11] had presented evidence of an association that the rate of half-pin site infection was significantly higher in half-pin fixators than in hybrid fixators. Thus, a keen sense of the above patients' characters and minutely changes around the screws is necessary for orthopedic surgeons to make a correct and quick response for the prevention of PTI. For instance, regular pin care can do a great favor, such as cleaning the pin tract with triple antibiotic ointment twice a day [19], changing dressing with chlorhexidine once a week, or let the wound uncovered. If the treatment is not done promptly, the pin tract may in danger of inflammation in which bacteria breed, and additional revision surgeries were brought to patients eventually.

According to the natural disadvantage of this technique, researches on PTI during bone transport therapy is urgently needed to avoid the horrible results described above and to give orthopedic surgeons a helpful favor. Therefore, the purpose of our study was to evaluate the incidence of PTI and preoperative or postoperative risk factors after external fixation treatment. This can also give an indication indirectly of the approximate distribution of PTIs in patients treated with an external fixator (including the type of modification treatment).

## Methods

After approval by the ethics committee of our institution, a retrospective evaluation of 130 patients (130 limbs) treated by the same surgery team for bone defects of the lower limb between January 2011 and February 2019 was conducted in this study. Trauma (62 cases), tumor resection (17 cases), and debridement of osteomyelitis (51 cases) were the most common reasons for bone defects. There were inclusion criteria that age older than 20 years, bone defects at least 4 cm of the lower limb, good compliance, and permission to the bone transport surgery. Others older than 60 years, poor compliance, or with any diseases involving the PTI (such as dermatitis, tuberculosis, autoimmune diseases, and metal allergy) were excluded. All bone defects of the lower limb were managed bone transport surgery using unilateral external fixation (Orthofix, Verona, Italy). Single-level bone transport was managed for bone defects ranging from 4.5 cm to 6 cm, and double-level bone transport was applied for more than 6 cm. This study complied with the Declaration of Helsinki, and informed consent was obtained from each patient.

### Surgical technique

In the prior stage of bone transport, all necrotic and infected bone and soft tissues are removed from the hardware completely, debrided thoroughly. The bacterial culture and antibiotic sensitivity test were conducted in exudation, to instruct the surgeon to apply appropriate postoperative antibiotics. The defects were filled with antibiotic (5 g vancomycin cement per 40 g gentamicin) PMMA bone cement (Heraeus, Hanau, Germany). Afterward, preoperative anteroposterior and lateral X-rays were used to evaluate the size of the defect and plan the structure of the external fixator. Bone transport surgery could be performed after the infection was controlled completely, which could be determined by laboratory parameters such as white blood cells (WBC), CRP (C-reactive protein), and ESR (erythrocyte sedimentation rate).

When the above preoperative preparation was achieved, we believe that the infection had been successfully eliminated and bone transport surgery was ready to perform. According to the preoperative plan, the external fixator of screw hole placement sleeve on the lateral side of the femur or the medial side of the tibia was placed. Under X-ray perspective, the external fixator was adjusted to the level of the coronal plane of the tibial or femoral force line, and the sleeve position on the skin was marked. Respectively, three stainless steel Schanz screws (4.5 mm threads, non-self-tapping Schanz screws) were implanted at the proximal and distal metaphysis of the tibia or femur with the aid of a sleeve, according to the position of the markers. And two Schanz screws (4.5 mm threads, non-self-tapping Schanz screws) were implanted at the bone transport segments. Ensure that each screw passes through both cortical layers. Hydroxyapatite-coated screws were applied if patients with osteoporotic. The external fixator sliding module was assembled and ensured that the sliding module can move up and down the track. Under X-ray perspective, the Schanz screws were adjusted to the best position respectively and tighten the external frame. Afterward, a minimally invasive osteotomy was performed using a Gigli saw was performed at the proximal and distal metaphysis of the tibia or femur to obtain a good blood supply. The osteotomy line of double-level bone transport was designed to the transport bone segment, ensuring that two Schanz screws can be inserted into each transport bone segment. An X-ray radiograph was arranged on the second postoperative day and intravenous antibiotics were conducted preventively for 3 days.

### Postoperative management

Bone transport started after a latent period of seven days. The proximal fragment and the distal fragment were distracted four times per day at a rate of 1 mm/day (single-level) or 2 mm/day (double level) retrospectively until the two fragments converged. Paid more attention to the pin tract care was recommended, washed the pin tract daily using an alcohol swab with a mass percentage of 75%; the patient was encouraged to carry out weight-bearing activities on crutches 1 week after surgery. Subsequently, radiographic examination, WBC, ESR, and CRP were examined at 1, 3, 6, 9, 12, 18, and 24 months after bone transport.

Non-weight-bearing ambulation started the day after surgery among all patients. Standardized pin care and observation were initiated immediately. The rhythm adapted to the patient’s tolerance and the quality of bone regeneration. Finally, the external fixator was removed when radiographs showed dense regenerated bone formation in the distraction osteogenesis area and connected to the docking site (3/4 of the total cortex). Meanwhile, detailed pin tract care instructions were provided to the patient, such as keeping clean and dry, precluding any distortion, curve, or contaminated stuff contacting with the pin tract. Additionally, patients were followed up biweekly initially, then monthly by the same senior orthopaedic surgeon until the external fixator was removed. Grading of the degree of PTI was based on the presence or absence of erythema, purulent discharge, and radiological evidence of pin loosening.

Briefly, postoperative pin tract care was performed by cefuroxime sodium mixed with 100 ml 0.9% saline for antibacterial treatment twice a day regularly. Treatment such as removing the external fixation, replacing the fixed pin tract, and dressing change frequently were prepared to conduct during distraction osteogenesis, preventing deep infection of the enhanced dressing infection. At the same time, hydroxyapatite pins were utilized near the joint, to take precautions for potential complications such as loosening of the pin path in patients with osteoporosis.

### Data collection

The demographic was recorded, such as age, gender, body mass index [BMI = weight (kg)/height (m^2^)], education, occupation, living environment, unhealthy habits (smoking and alcohol), comorbidities (hypertension and diabetes), type of bone transport (single level or double level), duration of disease, and the location of the bone defect.

Postoperative records included the time to bone union, external fixation time (EFT), and external fixation index (EFI) was recorded. Also, the marks of infection, like the skin temperature, white blood cell count, venous blood bacterial culture result, and pin tract secretion bacterial culture result, the inflammatory index was used to evaluate the grade of PTI. The Saw et al. classification [20] of PTI was applied in our study due to its simplicity and effectiveness. The details were presented in Table 1. Some treatment options were also recommended for the corresponding grading of PTI. For instance, the dressing change should be increased to twice a day in grade 1 and susceptible antibiotic combination therapy was given based on bacterial culture results of secretions in grade 2. The above treatment effect was poor when grade 3 PTI occurred. Thus, the location of the infection pin should be replaced in time and supplemented with effective antibacterial drugs to avoid the aggravation of infection.

**Table 1:** Grading system for pin tract infection based on clinical appearance

Grade	Description
Grade 1	Presence of either skin erythema or purulent discharge
Grade 2	Presence of both skin erythema and purulent discharge
Grade 3	Grade 2 symptoms and radiological evidence of osteolysis at bone interface

### Potential risk factors

Continuous variables included age, defect size (DS), the duration of initial disease, time to bone union, external fixator time (EFT), and external fixator index (EFI). And gender, body mass index (normal weight = BMI < 25 kg/m^2^, overweight = BMI > 25 kg/m^2^), living environment (urban or non-urban), education (primary, middle, or university), occupation (agriculture or non-agriculture), location of bone defect (femur or tibia), comorbidities such as diabetes, hypertension (yes or no), and the type of bone transport (single level or double level) were attributed to the categorical variables.

### Statistical analysis

The statistical analysis of data was calculated by an independent statistician using the SPSS 23.0 (IBM Corp, Armonk, NY, USA). The infection rate was analyzed and expressed as a percentage of the total individuals. Variables of the category, such as gender (male or female), living environment (urban or non-urban), education (primary, middle, university), occupation (agricultural or non-agricultural), smoking (yes or no), alcohol (yes or no), hypertension (yes or no), diabetes (yes or no), BMI > 25 (yes or no), type of bone transport (single-level or double-level), location (femur or tibia), were analyzed by the Pearson’s Chi-square test or Fisher exact test. Quantitative variables, included age, defect size (DS), the duration of initial disease, time to bone union, external fixator time (EFT), and external fixator index (EFI) were described with mean and standard deviation and analyzed by the *T*-test.

The variable with a P-value of 0.05 or less in the Pearson’s Chi-square test, Fisher exact test, or *T*-test was entered in the multivariate logistic regression model to assess the relationship between the explanatory variable and the grade of PTI. The odd ratio provides a 95% confidence interval and P-value. Statistical significance was P < 0.05.

## Results

The study evaluated the bone defects (59 femurs and 71 tibias) with a mean of 5.85 cm (4.2–7.4 cm) in a total of 130 patients (61 females, 91 males) treated by bone transport using a unilateral external fixator. The mean of previous surgical treatments in our cohort was 2.3, and patients with PTI received an average of 3.9 preoperative surgical treatments. There were 78 cases managed by single-level bone transport and 52 cases of double-level bone transport. A total number of 1082 Schanz screws were inserted into 130 limbs. None of the pins were buried, bent, or broken in the deep soft tissue during the whole treatment.

Among these, fifty-eight patients (44.6%) had varying degrees of PTI symptoms (thirty-nine classified as Saw’s grade 1, seventeen in grade 2, and two in grade 3) with an average follow-up time of 2.3 years, range 1.4–3.9 years. According to the results of infectious secretion culture, the bacteria of infection were common with Staphylococcus epidermidis (31 cases), followed by Staphylococcus aureus (19 cases), Micrococcus (6 cases), and Bacillus (2 cases) [10]. Pin loosening or deep PTI happened to 14 screws of nine patients. The symptoms were improved successfully by using sensitive intravenous antibiotic therapy to assist in replacing the pin tract. Besides, seven axial deviations were corrected by adjusting the external fixator manually. Eleven delayed unions on docking sites and two poor regenerate consolidations were recovered through surgical treatment of autologous bone grafts.

There was no significant difference concerning the age, education level, occupation, and defect size from the original cohort by the analysis of demographic data (P > 0.05). Conversely, gender, BMI, living environment, smoking and alcohol, comorbidities (such as hypertension, diabetes), duration of initial disease, time to bone union, EFT, EFI, type of bone transport, and location of bone defect were statistically significant (P < 0.05). Details were presented in Table 2.

**Table 2:** Bivariate analysis of patients (n = 130)

Variable	PTI n = 58	No PTI n = 72	t/*χ*^2^	p-value
Age, mean ± SD (years)	44.02 ± 7.18	42.45 ± 7.23	2.239	0.216
Male (%)	49 (84.4)	42 (58.3)	12.216	0.048
Living environment (%)				
Urban	47 (18.9)	27 (37.5)	5.517	< 0.001
Non-urban	11 (81.0)	45 (62.5)		
Education (%)				
Primary	35 (60.3)	27 (37.5)	0.592	0.014
Middle	19 (32.7)	22 (30.5)		
University	4 (6.8)	23 (31.9)		
Occupation (%)				
Agricultural	46 (79.3)	45 (62.5)	1.033	0.002
Non-agricultural	12 (20.6)	27 (37.5)		
Smoking yes (%)	43 (74.1)	37 (51.3)	2.061	0.039
Alcohol yes (%)	26 (44.8)	21 (29.1)	17.437	< 0.001
Hypertension yes (%)	37 (63.7)	23 (31.9)	7.437	< 0.001
Diabetes yes (%)	35 (60.3)	24 (33.3)	7.594	< 0.001
BMI > 25 (%)	33 (56.8)	13 (18.0)	2.034	0.042
Duration of initial disease, mean ± SD(month)	26.87 ± 8.02	23.61 ± 7.08	0.818	0.021
DS, mean ± SD (cm)	6.21 ± 1.33	5.49 ± 1.30	0.940	0.462
Time to bone union, mean ± SD (month)	8.21 ± 0.50	7.13 ± 0.58	2.752	0.034
EFT, mean ± SD (month)	9.76 ± 0.34	8.23 ± 0.61	3.668	0.002
EFI, mean ± SD (months/cm)	1.97 ± 0.31	1.52 ± 0.33	2.912	0.046
Type (%)				
Single-level	31 (53.4%)	47(65.2%)	2.239	0.025
Double-level	27 (46.5%)	25(34.7%)		
Location (%)				
Femur	35 (60.3%)	24 (33.3%)	12.216	< 0.001
Tibia	23 (39.6%)	48 (66.6%)		

*PTI* pin tract infection, *DS* defect size, *EFT* external fixation time, *EFI* external fixation index

Male, non-urban living, lower education level, agricultural work, smoking, diabetes, alcohol, hypertension, duration of initial disease > 24 months, BMI > 25, EFT > 9 months, EFI > 1.8 months/cm, double-level bone transport, defect of femur were significantly associated with the incidence of PTI by binary logistic regression analysis (Table 3). Time to bone union > 9 months was not significantly associated with PTI.

**Table 3:** Binary Logistic Regression analysis of risk factors in pin tract infection

Risk factor	Wald	P	Odds ratio (CI)
Male	4.275	0.028	1.71 (1.02–2.31)
Non-urban living	8.697	0.002	1.75 (1.24–3.26)
Lower education	5.339	0.009	1.10 (0.78–1.51)
Agricultural work	0.843	0.032	1.86 (0.94–2.39)
Smoking	2.980	0.070	1.53 (0.76–1.89)
Alcohol	2.938	0.036	1.07 (0.81–1.64)
Hypertension	6.834	0.007	1.49 (0.89–1.97)
Diabetes	6.609	0.003	1.26 (1.12–2.64)
BMI > 25	5.560	0.018	1.14 (0.94–1.43)
Duration of initial disease > 24 months	5.213	0.042	1.02 (0.89–1.25)
Defect of femur	2.438	< 0.001	1.06 (0.61–2.31)
Time to bone union > 9 months	1.291	0.916	0.33 (0.06–0.87)
EFT > 9 months	4.637	< 0.001	0.81 (0.24–1.33)
EFI > 1.8 months/cm	4.892	0.012	0.97 (0.65–1.43)
Double-level bone transport	2.735	< 0.001	0.18 (0.02–0.36)

*DS* defect size, *EFT* external fixation time, *EFI* external fixation index

**Fig. 1:**
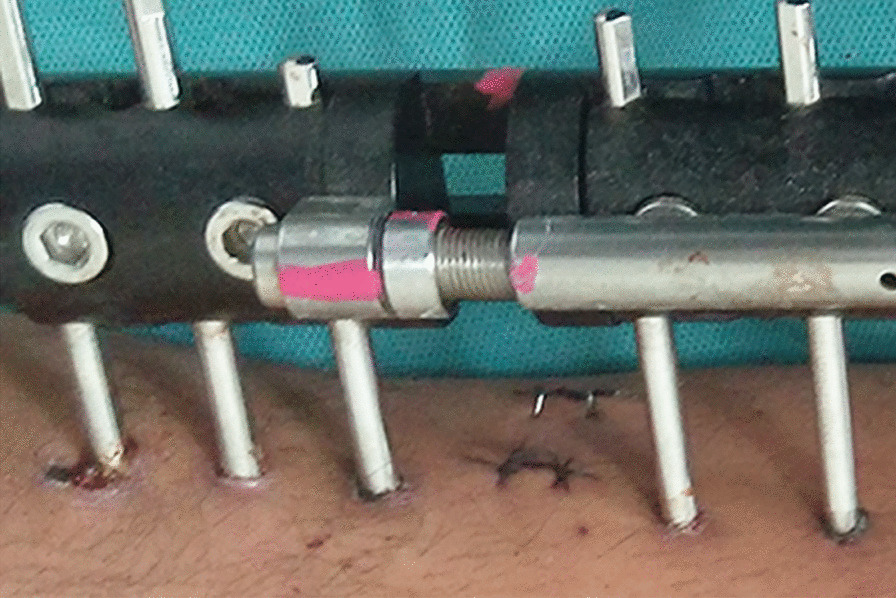
Grade I infection. There is a purulent discharge around the first proximal pin tract

**Fig. 2:**
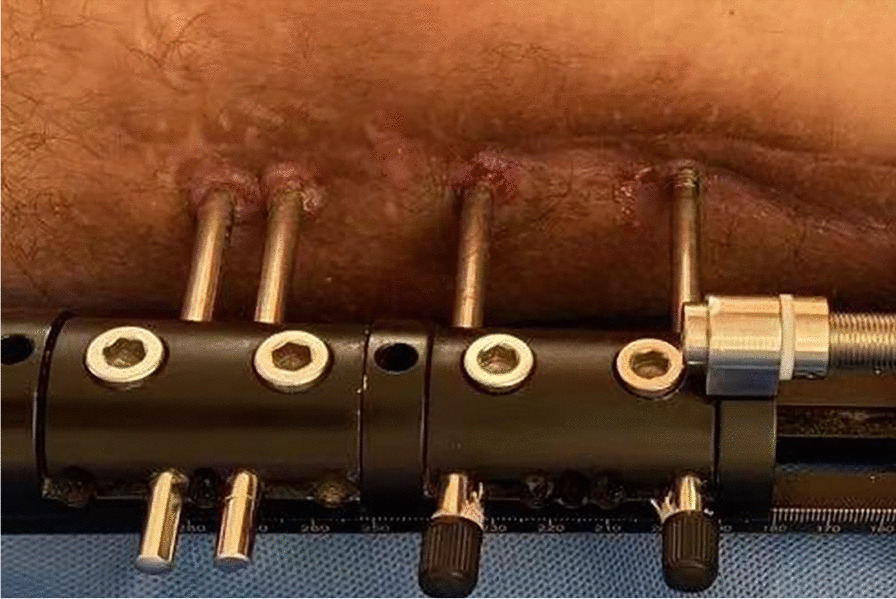
Grade II infection. There are both skin erythema and purulent discharge around the pin tract

**Fig. 3:**
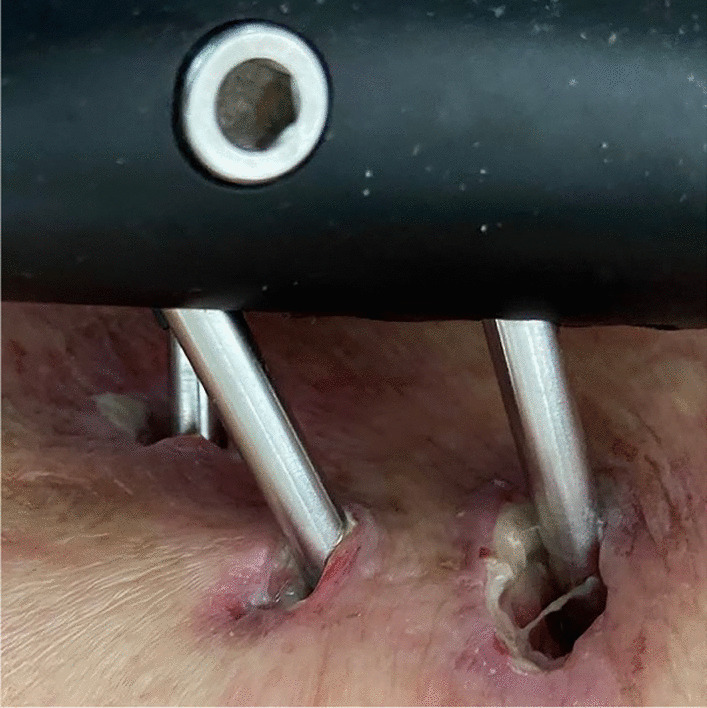
Grade II infection. There are both skin erythema and purulent discharge around the pin tract

Male, non-urban living, agricultural work, smoking, diabetes, BMI > 25, EFI > 1.8 months/cm, double-level bone transport, defect of femur were independently associated with malleolar OA on multivariate logistic regression analysis and constituted Table 4. The top five risk factors were male (84.4%), non-urban living (81.0%), agricultural work (79.3%), smoking (74.1%), and diabetes (60.3%). The typical appearance pictures of the PTI were placed in Figs. 1, 2, 3.

**Table 4:** Multivariable Logistic Regression analysis of risk factors in pin tract infection

Risk factor	Wald	P	Odds ratio (CI)
Male	4.275	0.021	1.71 (0.87–1.11)
Non-urban living	8.697	0.002	1.75 (0.36–1.89)
Lower education level	5.339	0.651	0.23 (0.05–0.61)
Agricultural work	0.843	0.008	1.86 (1.23–2.12)
Smoking	2.980	0.070	1.53 (1.29–1.91)
Alcohol	2.938	0.843	0.31 (0.07–0.67)
Hypertension	6.834	0.714	0.11 (0.04–0.38)
Diabetes	6.609	0.003	1.52 (1.02–1.83)
BMI > 25	5.560	0.018	1.14 (1.03–1.83)
Duration of initial disease > 24 months	6.122	0.627	0.64 (0.31–0.97)
EFT > 9 months	5.638	0.261	1.13 (0.78–1.64)
EFI > 1.8 months/cm	2.394	0.032	0.67 (0.32–1.02)
Double-level bone transport	2.735	< 0.001	0.18 (0.05–0.31)
Time to bone union	5.317	0.382	0.44 (0.08–0.73)
Defect of femur	2.438	0.024	1.06 (0.61–1.27)

*DS* defect size, *EFT* external fixation time, *EFI* external fixation index

## Discussion

Nearly a decade of development, bone transport using unilateral external fixators based on the Ilizarov technique has become a practical tool in the orthopaedic surgeon’s arsenal for the treatment of bone defects in the lower limb [1, 10, 13, 16, 18, 21]. However, one of its natural drawbacks is that the utilization of external fixation requires exposure to the external environment, which poses a risk of PTI during treatment [22–25]. This is an undoubted obstacle that should not be underestimated for the widespread use of external fixation techniques. Therefore, this study evaluated the epidemiological and therapeutic risk factors of PTI and provides solutions for improving the safety of external fixation treatment.

According to this study, the phenomenon that PTI rate was close to 44.6% for the number of patients. Interestingly, the incidence of PTI also fluctuates greatly in different research. Ten cases (7.3%) of PTI, two of which developed osteomyelitis was reported by Botte et al. [26] in 137 patients with hand or wrist fracture or dislocation treated with external fixation. Stahl and Schwartz [27] reported PTIs happened to 13 of 236 patients (5.5%), including one case of osteomyelitis. PTI in 19 of 189 (10%) patients who underwent external fixation, of which 1 required drainage for deep infection was observed by Hsu et al. What’s more, Rommen et al. [28] reported 9.4% mild and 4.0% severe PTI [29]. A deep infection rate of 8.0% was noticed by Ktistakis et al. [29]. Both the above and our study showed that a large proportion of patients had a chance to PTI when their bony defects or nonunion were treated by the external fixator. However, PTIs in our cohort was higher than reported in most of the published studies. The patients in our cohort with PTI have a long history of osteomyelitis mostly than patients without PTI (P < 0.05). Furthermore, patients with PTI received an average of 3.9 preoperative surgical treatments, which is more than the whole average. This permitted the incidence of disuse osteoporosis higher, which may lead to loosening of the pin tract. At the same time, the microorganisms parasitized around the bone defect for a long time, which may lead to scarring of the surrounding connective tissue or even inflammatory granulomas [30, 31]. We speculated that the holding force at the screws after bone transport surgery may be weakened by above, which may be more likely to be infected and damaged by microbial breeding, and increased the chance of PTI. Hence, for these patients, the external frame was designed preoperatively to implant screws in normal skin as much as possible, or bone transport surgery was performed after the skin condition in the bone defect area improved by flap surgery. Fortunately, most PTI after bone transport surgery were noted as superficial, and satisfactory results can be obtained by oral antibiotics or strengthened pin tract care.

Among 58 cases of PTI in the period of bone transport treatment over periods of 1.4–3.9 years, the rate of PTI in the male patients (OR 1.71, CI 1.02–2.31) was higher than the females were observed in the study. Further, it was noticed that those who live in a non-urban city (OR1.75, CI1.24–3.26), with a low level of education (OR 1.10, CI 0.78–1.51), unhealthy habits [smoking (OR 1.53, CI 0.76–1.89), alcohol (OR 1.07, CI 0.81–1.64)], BMI > 25 (OR 1.14, CI 0.94–1.43), and chronic diseases [such as hypertension (OR 1.49, CI 0.89–1.97) and diabetes (OR 1.26, CI 1.12–2.64)], are more likely to develop PTI (P < 0.05). As generally accepted, living in communities with poor sanitation are more likely to be exposed to bacteria and other microorganisms. The same situation was observed by Saw et al. Moreover, patients with low education are easier to develop unhealthy lifestyle habits, which will also promote the occurrence of PTI significantly. The study published by Edwards et al. [32] considered that patients with poor living habits and compliance are likely to obtain external fixation complications, such as PTI. Besides, our study showed that the risk of PTI might be reduced for patients without bad habits or comorbidities (e.g., hypertension, diabetes). Therefore, a good follow-up mechanism, such as a web follow-up questionnaire, was recommended to establish for patients with the above risk factors. Simultaneously, it was also practical to prophylactically increase the frequency of dressing change in the pin tract of these patients (every 2 days). In detail, the pin tract was wiped carefully and thoroughly by a cotton swab moistened with sterile 0.9% saline. The patients were encouraged to practice self-cleaning of the pin tract under the supervision of a clinician and were not allowed to be discharged until determined to be competent for this technique. Also, it was of great benefit for the prevention of PTI by helping them quit bad living habits.

The phenomenon of exposure to occupational factors for PTI is also not negligible, especially for agricultural practitioners in developing areas. The workload of heavy physical strength, the working environment of complex species of microorganisms, and work-related high-pressure psychological state may be associated with a high incidence of infectious diseases [12, 29, 33, 34]. In this study, the rate of PTI was 35.4% in patients working in agriculture approximately. Additionally, the bacterial culture test results of these patients with PTI were mainly Staphylococcus epidermidis and Staphylococcus aureus. We considered that most of these patients lived in non-urban areas, without experiencing high education and processing healthy living habits (smoking, alcohol, and so on). Simultaneously, these health-damaging lifestyle habits may breed more comorbidities, which led to vulnerable body immunity and infection resistance. The postoperative follow-up guidance treatment may then not receive the patients' good compliance, which promoted the occurrence of PTI. Hence, it was of great importance of introducing the treatment process of bone transport to the patient in detail, and increasing the patient's attention to postoperative pin tract care was helpful to prevent PTI, particularly for patients with the above risk factors.

With a prolonged treatment period of external fixation of bone transport, the cortical bone of the diaphysis stressed by providing stability becomes cancellous. This resulted in a decrease in the bending strength of the extended bone and an increased risk of PTI. Similarly, the incidence of PTI was lower in single-level bone transport than double-level bone transport (P < 0.05). The direct reason for this occasion is that larger bone defects, especially more than 6 cm, required double-level bone transport to shorten the whole treatment time. Inevitably, the double-level bone transport (OR 0.18, CI 0.02–0.36) adds the number of the pins and the velocity of distraction osteogenesis. The EFT (OR 0.81, CI 0.24–1.33), and EFI (OR 0.97, 0.65–1.43) were raised further. These giving the skin greater tension, which can expose more subcutaneous tissue to the air easily and prolong the time for transport area mineralization, resulting in a higher potential for PTI. A study evaluating the clinical efficacy of double-level bone transport in sixteen large post-traumatic bone defects noticed a similar distribution of complications [1]. Besides, the study published by Bezstarosti et al. [2] also clarified a fact that the incidence of postoperative complications is related to the extension of external fixation time in a meta-analysis. When double-level bone transport was selected for the treatment of bone defects, the axial load of long bones during the distraction stage was also more than that of single-level bone transport. Therefore, the external frame should be designed to be parallel to the coronal plane of long bones, to avoid damage or exposure to the soft tissues around the pin tract due to the unbalanced force when distracting the transport bone segments. In our experience, three Schanz screws used for fixation in the proximal and distal metaphysis respectively, and two used for fixation in each transported bone segment can achieve the above framework satisfactorily. Certainly, cancellous screws should be applied in the metaphysis, and cortical screws in the mid-shaft bone to allow better axial holding force of the external fixator. It was also important to pass the screws with the aid of a sleeve to avoid necrosis by entangling the subcutaneous soft tissue. Simultaneously, the screws should be passed through the two cortex perpendicular to the medullary cavity to ensure the screws’ holding force for the prevention of loosening and infection. It was recommended that the surgical procedure was conducted by a team experienced in bone transport using an external fixator to avoid repeated intraoperative pinning, which may increase the risk of PTI. Besides, the choice of minimally invasive osteotomy using Gigli saw was recommended since its ability to avoid thermal damage mostly to bone and soft tissue, which may lead to infection of the subcutaneous cavity. Furthermore, since the muscle and soft tissue coverage of the femur are richer than that of the tibia, the resistance encountered during the bone transport requires a longer external fixation time. This directly explains the higher incidence of PTI on the femoral bone transport than the tibia (P < 0.05). The same phenomenon was observed by Inan et al. [35]. Thus, longer antimicrobial treatment and more frequent pin tract care are recommended when bone transport applying to the femur.

The average PTI per patient consists of 44.6% (67.2% grade 1, 29.3% grade 2, and 3.4% grade 3) in our study. The higher rate of PTI may be the result of a state of the body stress response as well [36]. With the decline in autoimmunity potentially and the abused use of multiple antibiotics, the risk of infection is brought in further largely [37]. In our research, patients with diabetes or hypertension may be inhibited in partial response segments to invading microorganisms, including neutralizing toxins, phagocytosis, and bactericidal effects. Another speculation of us, the high incidence of PTI in patients in our research may be the immune rejection of the implanted stainless steel screws. The chance of an inflammatory response in the internal environment surrounding the screws was then increased. In response to such phenomena, the biocompatibility of titanium screws is better than traditional stainless steel screws were disclosed by more and more researchers [38, 39]. Thus, there is a trend that the use of titanium alloy screws has become more prevalent for future treatment with external fixation.

With these, the most straightforward risk factors to the PTI can be obtained from our research. To our knowledge, there is a large number of benefits to highlight these high-risk factors to avoid PTI feasibly. One of them is helping patients stay away from risk factors as soon as possible, which can effectively prevent bacteria from contaminating the pin tract. Sure, the correct pin tract care is the important content of patient postoperative care.

Several limitations occurred in this study. First of all, an objective attitude should be taken towards the interpretation of the risk factors predicted by our study, because of the smaller sample size of PTI. Secondly, due to the follow-up time, risk factors related to PTI may not be reliably documented. Last, our study focused only on lower limb bone transport, showing results and complications in a single center and a single surgeon case series at long-term follow-up.

## Conclusion

In short, male, non-urban living, agricultural work, smoking, and diabetes were the top five significant risk factors. Awareness of predictable risk factors of PTI in bone transport is beneficial to avoid or detect serious complications early, thereby improving the therapeutic effect.

## Data Availability

The datasets analyzed during the current study are available from the corresponding author on reasonable request.
